# Association between dietary magnesium intake and all-cause mortality in patients with chronic kidney disease: Insights from NHANES 1999–2018

**DOI:** 10.1016/j.clinme.2026.100633

**Published:** 2026-08-08

**Authors:** Zi-yan Xu, Jing Zou, Li-jun Xie, Ying Ye, Kai-ying He, Juan Zhu, Xiao-lan Wang, Ruo-li Wang, Yi-jia Luo, Jian-hui Zhang, Qian Chen, Dan-dan Ruan, Yan-feng Zhou, Mei-zhu Gao, Li Zhang, Yun-fei Li, Zhu-ting Fang, Fang-meng Huang, Bin Hu, Jie-wei Luo, Zhi-hai Zheng, Li Chen

**Affiliations:** a Department of Traditional Chinese Medicine and Pediatrics and Nephrology, Shengli Clinical Medical College of Fujian Medical University, Fuzhou University Affiliated Provincial Hospital, Fuzhou, China; b Department of Oncology, Zhangzhou Traditional Chinese Medical Hospital, Affiliated Zhangzhou TCM Hospital, Fujian University of Traditional Chinese Medicine, Zhangzhou, China; c Fujian University of Traditional Chinese Medicine, Fuzhou, China; d Department of Emergency, Fuzhou University Affiliated Provincial Hospital, Fuzhou University, Fuzhou, China; e Fujian Provincial Key Laboratory of Emergency Medicine, Fujian Provincial Institute of Emergency Medicine, Fujian Emergency Medical Center, Fuzhou, China; f Department of Oncology and Vascular Interventional Therapy, Clinical Oncology School of Fujian Medical University, Fujian Cancer Hospital (Fujian Branch of Fudan University Shanghai Cancer Center), Fuzhou, China; g Department of Cardiology, Beijing Anzhen Hospital, Capital Medical University, Beijing Institute of Heart Lung and Blood Vessel Disease, Beijing Key Laboratory of Precision Medicine of Coronary Atherosclerotic Disease, Clinical Center for Coronary Heart Disease, Capital Medical University, Beijing, China; h Fujian Provincial Hospital, Fuzhou University Affiliated Provincial Hospital, Fuzhou University, Fuzhou, China

**Keywords:** Magnesium, Dietary, Chronic kidney disease, All-cause mortality, NHANES

## Abstract

**Background:**

We investigated the association between food-derived dietary magnesium intake and all-cause mortality among adults with chronic kidney disease.

**Methods:**

We analysed data from adults aged ≥20 years with CKD who participated in the National Health and Nutrition Examination Survey between 1999 and 2018. Mortality status was ascertained through linkage to the National Death Index through 31 December 2019. Multivariable Cox proportional hazards models were used to evaluate the association between dietary magnesium intake and all-cause mortality. Restricted cubic spline analysis, Kaplan–Meier survival analysis, subgroup analyses, and sensitivity analyses were also performed.

**Results:**

A total of 8,104 CKD patients were included in this cohort study with a median follow-up of 85 months, during which 3,134 (38.7%) died from all causes. In the fully adjusted Cox model, each 100 mg/day increase in dietary magnesium intake was associated with a 7.5% lower risk of all-cause mortality (HR = 0.925, 95% CI 0.881–0.971; P = 0.002). Compared with the lowest quartile, adjusted hazard ratios were 0.978 (95% CI 0.883–1.083) for Q2, 0.904 (95% CI 0.804–1.016) for Q3, and 0.866 (95% CI 0.744–1.007) for Q4, with a significant trend across quartiles (P for trend = 0.036). RCS analysis demonstrated a significant overall association with no evidence of nonlinearity (P for overall association = 0.009; P for nonlinearity = 0.472). Kaplan–Meier analysis showed higher survival probabilities among participants with higher dietary magnesium intake.

**Conclusions:**

Higher dietary magnesium intake was independently associated with a lower risk of all-cause mortality among adults with CKD. These findings suggest that adequate dietary magnesium intake may represent an important nutritional factor associated with prognosis in CKD. Further prospective studies and randomised clinical trials are warranted to determine whether increasing dietary magnesium intake can improve clinical outcomes.

**Summary statement:**

Higher dietary magnesium intake was independently associated with lower all-cause mortality among patients with chronic kidney disease in NHANES 1999–2018. These findings highlight the potential protective role of adequate magnesium intake in improving long-term survival in this population.

## Highlights

• This study included 8,104 adults with CKD from NHANES 1999–2018, with a median follow-up of 85 months.
• Higher dietary magnesium intake was associated with lower all-cause mortality in adults with CKD.
• Each 100 mg/day higher magnesium intake was associated with a 7.5% lower adjusted mortality risk.
• The dose–response association was approximately linear, with no significant evidence of nonlinearity.
• Findings were generally consistent in sensitivity analyses, supporting further prospective research.

## Introduction

Chronic kidney disease (CKD) represents a substantial and growing global health burden. According to the Global Burden of Disease Study 2023, an estimated 788 million adults aged 20 years and older were living with CKD worldwide in 2023, corresponding to a global age-standardised prevalence of 14.2%.^1^ CKD was the ninth leading cause of death globally and accounted for approximately 1.48 million deaths in 2023. Beyond its direct mortality burden, CKD may progress to kidney failure and is an important contributor to cardiovascular morbidity and mortality. Therefore, identifying potentially modifiable factors associated with long-term outcomes is important for reducing the growing clinical and public health burden of CKD.

Magnesium is an essential mineral and one of the most abundant intracellular cations in the human body. It plays a crucial role in various physiological processes, including DNA and protein synthesis, cellular energy metabolism, and nerve conduction.^2^, ^3^ CKD patients are often at risk for multiple nutritional disorders, such as protein-energy malnutrition, micronutrient deficiencies, and electrolyte disturbances.^4^ Magnesium homoeostasis in CKD is complex and may be influenced by factors other than dietary intake. Although reduced kidney function alters renal magnesium excretion, tubular dysfunction, tubulopathies, and proteinuria may impair tubular magnesium reabsorption and promote renal magnesium wasting. Diabetes mellitus and medications that affect intestinal magnesium absorption or renal magnesium handling may further modify magnesium status. Consequently, dietary magnesium intake may not directly reflect circulating magnesium concentrations in patients with CKD. Hypomagnesaemia is common in CKD patients,^5^ and several studies have demonstrated an independent association between low serum magnesium levels and increased all-cause mortality in CKD patients.^6^, ^7^, ^8^, ^9^ Dietary intake is the primary source of magnesium. Dietary magnesium is obtained primarily from plant-based foods, including green leafy vegetables, whole grains, legumes, nuts, and seeds. Some evidence suggests that changes in crop varieties, soil mineral availability, intensive agricultural practices, and food processing may have contributed to reductions in the magnesium content or availability of some fruits and vegetables.^10^ Although magnesium may also be obtained from dietary supplements, the present study specifically evaluated food-derived magnesium obtained from foods and beverages and did not include magnesium from dietary supplements. Previous studies showed that dietary magnesium deficiency was significantly associated with all-cause and cardiovascular mortality in elderly hypertensive patients.^11^ Moreover, higher magnesium intake was linked to a reduced risk of abnormal glucose metabolism and decreased progression from prediabetes to diabetes.^12^ In other conditions, increased dietary magnesium intake was associated with lower all-cause mortality in arthritis patients,^13^ and animal studies showed that magnesium supplementation significantly reduced mortality in uraemic rats.^14^ However, evidence on the potential survival benefits of dietary magnesium intake in CKD patients remains limited.

This study aimed to investigate the association between dietary magnesium intake and all-cause mortality among adults with CKD using data from NHANES 1999–2018. We hypothesised that higher dietary magnesium intake would be associated with a lower risk of all-cause mortality.

## Methods

### Data source and study population

Data for this study were sourced from the NHANES, conducted by the National Center for Health Statistics (NCHS) to assess the health and nutritional status of the U.S. residents. NHANES utilises a complex, multi-stage stratified sampling design to ensure a representative sample of the U.S. population. Data collection includes two components: household interviews and medical examinations at mobile examination centres (MEC). The study protocol was approved by the NCHS Institutional Review Board, and written informed consent was obtained from all participants.

This study included adults aged ≥20 years with CKD who participated in 10 NHANES cycles between 1999 and 2018. CKD was defined as an estimated glomerular filtration rate (eGFR) <60 mL/min/1.73 m² and/or a urinary albumin-to-creatinine ratio (UACR) ≥3 mg/mmol (approximately ≥30 mg/g).^15^ Kidney function and albuminuria were assessed at a single examination because repeated measurements over at least three months were unavailable. Therefore, CKD chronicity could not be confirmed according to the clinical definition. Participants in eGFR categories G1 or G2 were classified as having CKD only when albuminuria was present.^16^

**Fig. 1:**
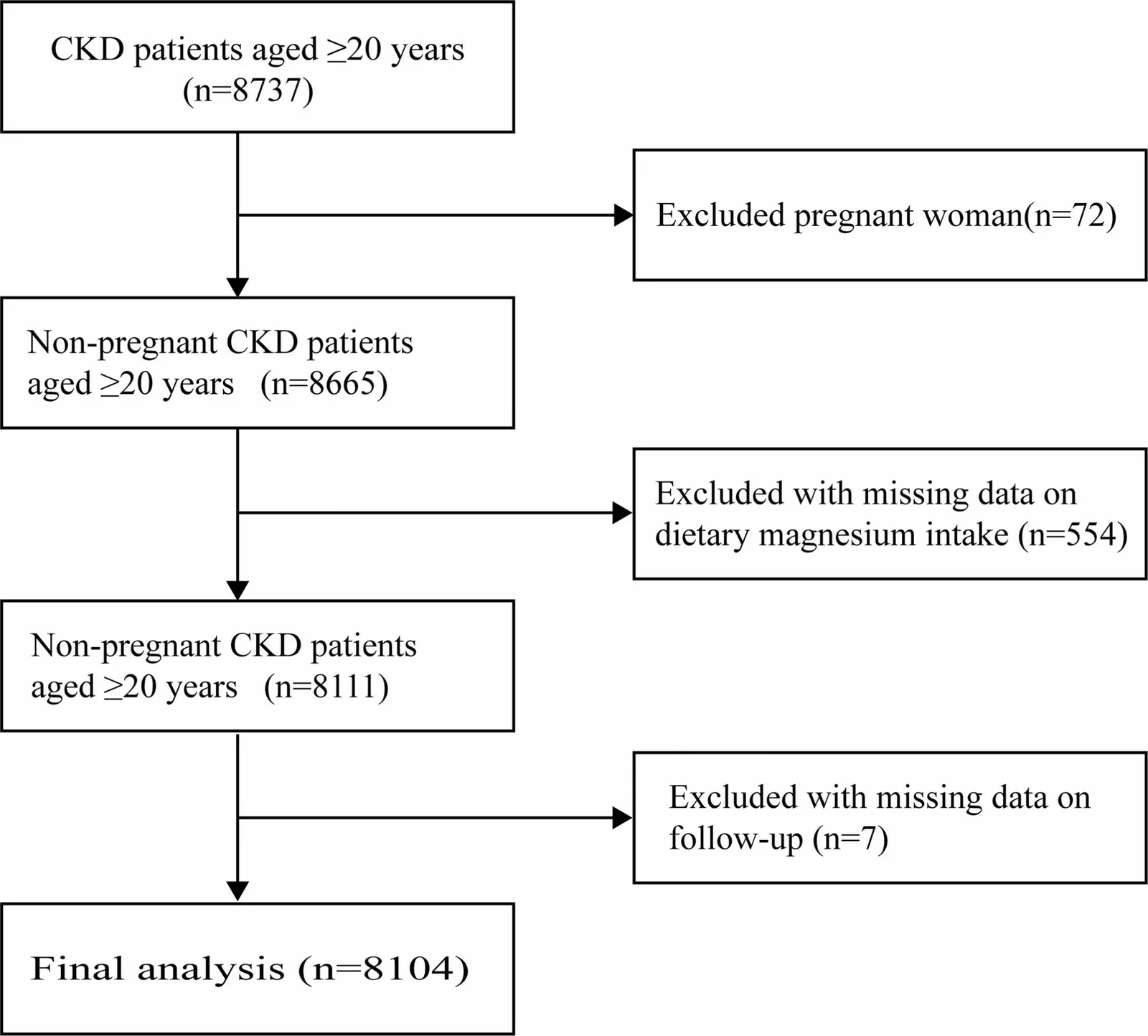
Flow chart of study participants.

The eGFR was calculated using the Chronic Kidney Disease Epidemiology Collaboration equation. UACR was calculated using urinary albumin concentration and urinary creatinine concentration. Participants were excluded if they were pregnant (n = 72), had missing dietary magnesium data (n = 554), or had missing mortality data (n = 7), leaving 8,104 participants for analysis (Fig. 1).

The eGFR categories were G1 (≥90 mL/min/1.73 m²), G2 (60–89 mL/min/1.73 m²), G3 (30–59 mL/min/1.73 m²), G4 (15–29 mL/min/1.73 m²), and G5 (<15 mL/min/1.73 m²). Albuminuria categories were A1 (<3 mg/mmol), A2 (3–30 mg/mmol), and A3 (>30 mg/mmol).

### Assessment of dietary magnesium

In the NHANES dietary survey, respondents were asked about the types and quantities of food and beverages consumed in the past 24 h. Between 1999 and 2002, dietary data were collected using the Computer Assisted Dietary Interview System (CADI), which employed a multiple-pass recall approach. From 2003 to 2004, dietary data collection transitioned to the Automated Multiple-Pass Method (AMPM) developed by the U.S. Department of Agriculture (USDA), a fully computerised recall technique. Two main data files were generated from the dietary interview dataset: the total nutrient intakes file, which includes total nutrient intakes, plain water intake, and the frequency of fish and shellfish consumption, and the individual foods file, which provides detailed information on individual foods reported by the respondents. Detailed dietary survey methods can be found in the Dietary Interviewer Procedures Manual (Https://wwwn.cdc.gov/nchs/nhanes/continuousnhanes/default.aspx). For this study, dietary magnesium data were sourced from the total nutrient intakes file, with magnesium intake measured in milligrams. The present analysis focused exclusively on magnesium obtained from foods and beverages during the first 24-hour dietary recall and did not include magnesium from dietary supplements.

### Ascertainment of mortality

The outcome of this study was all-cause mortality, sourced from the public-use mortality files provided by the NCHS. These files employ a probabilistic matching algorithm to link participant data with death certificate records from the Centers for Disease Control's National Death Index (NDI). Mortality data were updated through December 31, 2019. Causes of death were identified according to the International Classification of Diseases, 10th Revision (ICD-10), and all-cause mortality refers to the total number of deaths from any cause.^17^

### Assessment of covariates

Based on previous studies and the KDIGO 2024 Clinical Practice Guideline for the Evaluation and Management of Chronic Kidney Disease,^18^, ^19^, ^20^, ^21^ the covariates included in this study were: age, sex, marital status, race/ethnicity, educational level, family income, body mass index (BMI), smoking status, drinking status, physical activity, systolic blood pressure, diastolic blood pressure, haemoglobin, glycated haemoglobin (HbA1c), serum calcium, serum phosphorus, protein intake, total energy intake, carbohydrate intake, total fat intake, hypertension, diabetes, coronary heart disease, stroke, chronic obstructive pulmonary disease (COPD), cancer history, eGFR category, and urinary albumin-to-creatinine ratio (UACR) category.

Marital status was categorised as married/partnered (married or living with a partner) or other (widowed, divorced, separated, or never married). Race/ethnicity was categorised as non-Hispanic White, non-Hispanic Black, Mexican American, and other. Educational level was divided into less than nine years, 9–12 years, and more than 12 years. Family income was classified according to the poverty income ratio (PIR) as low (PIR <1.3), medium (PIR between 1.3 and 3.5), and high (PIR >3.5), as outlined in a US government report.^22^

Smoking status was defined as never smoked (less than 100 cigarettes in lifetime), former smoker (more than 100 cigarettes in lifetime but not currently smoking), or current smoker (more than 100 cigarettes in lifetime and smoking daily). Drinking status was classified as yes or no (defined as drinking more than 12 alcoholic beverages annually). Physical activity was categorised as <150 min/week or ≥150 min/week of moderate-intensity exercise. Systolic and diastolic blood pressures were taken as the average of multiple measurements. Laboratory biomarkers were measured according to the standardised, cycle-specific NHANES laboratory protocols. Protein intake, energy intake, carbohydrate intake, and total fat intake were obtained from the total nutrient intakes file in the dietary data. Definitions of stroke, hypertension, coronary heart disease, and diabetes were based on self-reports from the survey questionnaire. COPD was defined as a self-reported physician diagnosis of chronic bronchitis and/or emphysema. The eGFR and UACR categories were defined as described above.

### Statistical analysis

Participants were categorised into quartiles of dietary magnesium intake according to its distribution in the analytic sample, with the lowest quartile used as the reference group. Dietary magnesium intake was also analysed as a continuous variable, and hazard ratios were estimated for each 100 mg/day increment. Categorical variables are presented as numbers and percentages, whereas continuous variables are presented as means with standard deviations or medians with interquartile ranges, as appropriate. Differences across quartiles were evaluated using one-way analysis of variance for normally distributed continuous variables, the Kruskal–Wallis test for skewed continuous variables, and the chi-squared test for categorical variables.

Multivariable Cox regression models were used to estimate hazard ratios and 95% confidence intervals for the association between dietary magnesium intake and all-cause mortality. Model 1 was adjusted for age, sex, marital status, race/ethnicity, educational level, and family income. Model 2 additionally included body mass index, smoking status, drinking status, physical activity, systolic blood pressure, and diastolic blood pressure. Model 3 was further adjusted for haemoglobin, glycated haemoglobin, serum calcium, serum phosphorus, protein intake, total energy intake, carbohydrate intake, and total fat intake. Model 4 additionally included hypertension, diabetes, coronary heart disease, stroke, chronic obstructive pulmonary disease, cancer history, eGFR category, and UACR category.

Linear trends across magnesium intake quartiles were assessed by entering the quartile variable as an ordinal term in the fully adjusted Cox model. Kaplan–Meier survival curves were used to compare survival probabilities across magnesium intake quartiles, with differences assessed using the log-rank test. Restricted cubic spline models with four knots were fitted to evaluate the potential nonlinear association between dietary magnesium intake and all-cause mortality.^23^

Prespecified subgroup analyses were conducted according to age, sex, physical activity, diabetes, hypertension, and coronary heart disease. Effect modification was evaluated using likelihood ratio tests comparing models with and without interaction terms.

Sensitivity analyses were performed by excluding participants who died within the first two years of follow-up, participants with extreme daily energy intake (<500 or >5,000 kcal/day), participants identified as receiving dialysis, and participants with missing covariate data.

The proportional hazards assumption was evaluated using Schoenfeld residuals. Although no violation was observed for the dietary magnesium exposure variable, evidence of non-proportionality was detected for several covariates and for the global model; this was considered when interpreting the Cox regression estimates.

## Results

### Characteristics of the study participants

In this study, a total of 8,104 CKD patients were included. Table 1 summarises the characteristics of the CKD patients according to quintiles of dietary magnesium intake.

**Table 1:** Characteristics of chronic kidney disease patients stratified by quartiles of dietary magnesium intake.

Characteristic	Total	Dietary magnesium intake, mg/day				*p-*value
		Quartile 1 (≤169.5)	Quartile 2 (170.0–234.0)	Quartile 3 (235.0–323.0)	Quartile 4 (≥323.1)	
NO.	8,104	2,019	2,016	2,043	2,026	
Age, mean (SD), y	63.9 (16.6)	65.2 (16.5)	65.3 (16.0)	64.2 (16.7)	61.0 (16.8)	<0.001
Sex, male, n (%)	3,885 (47.9)	732 (36.3)	863 (42.8)	1,016 (49.7)	1,274 (62.9)	<0.001
Marital status, n (%)						<0.001
Married/Partnered	4,430 (54.7)	976 (48.3)	1,072 (53.2)	1,140 (55.8)	1,242 (61.3)	
Other	3,674 (45.3)	1043 (51.7)	944 (46.8)	903 (44.2)	784 (38.7)	
Race/ethnicity, n (%)						<0.001
Non-Hispanic white	4,103 (50.6)	947 (46.9)	1,028 (51.0)	1,075 (52.6)	1,053 (52.0)	
Non-Hispanic black	1,700 (21.0)	576 (28.5)	432 (21.4)	365 (17.9)	327 (16.1)	
Mexican American	1,218 (15.0)	265 (13.1)	283 (14.0)	321 (15.7)	349 (17.2)	
Others	1,083 (13.4)	231 (11.4)	273 (13.5)	282 (13.8)	297 (14.7)	
Education level, n (%), y						<0.001
<9	1,401 (17.3)	441 (21.8)	376 (18.7)	322 (15.8)	262 (12.9)	
9–12	3,301 (40.7)	950 (47.1)	806 (40.0)	805 (39.4)	740 (36.5)	
>12	3,402 (42.0)	628 (31.1)	834 (41.4)	916 (44.8)	1,024 (50.5)	
Family income, n (%)						<0.001
Low	2,737 (33.8)	847 (42.0)	688 (34.1)	609 (29.8)	593 (29.3)	
Medium	3,450 (42.6)	835 (41.4)	898 (44.5)	894 (43.8)	823 (40.6)	
High	1,917 (23.7)	337 (16.7)	430 (21.3)	540 (26.4)	610 (30.1)	
Smoking status, n (%)						0.266
Never	4,034 (49.8)	1,012 (50.1)	999 (49.6)	1,044 (51.1)	979 (48.3)	
Current	2,735 (33.7)	652 (32.3)	687 (34.1)	690 (33.8)	706 (34.8)	
Former	1,335 (16.5)	355 (17.6)	330 (16.4)	309 (15.1)	341 (16.8)	
Drinking, n (%)	5,339 (65.9)	1,169 (57.9)	1,282 (63.6)	1,358 (66.5)	1,530 (75.5)	<0.001
Physical activity, n (%)						<0.001
<150 min/week	5,128 (63.3)	1,401 (69.4)	1,355 (67.2)	1,282 (62.8)	1,090 (53.8)	
≥150 min/week	2,976 (36.7)	618 (30.6)	661 (32.8)	761 (37.2)	936 (46.2)	
BMI, mean (SD), kg/m²	30.0 (7.3)	30.1 (7.2)	30.1 (7.4)	29.8 (7.2)	30.0 (7.3)	0.651
SBP, mean (SD), mmHg	138.4 (27.3)	141.5 (29.0)	139.2 (28.4)	137.4 (26.1)	135.7 (25.2)	<0.001
DBP, mean (SD), mmHg	70.3 (18.4)	70.4 (19.7)	69.5 (18.3)	69.6 (18.6)	71.8 (17.0)	<0.001
HGB, mean (SD), g/dL	13.8 (1.7)	13.4 (1.7)	13.6 (1.7)	13.9 (1.6)	14.1 (1.7)	<0.001
HbA1c, mean (SD), %	6.3 (1.6)	6.2 (1.6)	6.3 (1.6)	6.2 (1.5)	6.3 (1.6)	0.457
Total Ca^a^, mean (SD), mmol/L	2.4 (0.1)	2.4 (0.1)	2.4 (0.1)	2.4 (0.1)	2.4 (0.1)	0.692
P^a^, mean (SD), mg/dL	3.7 (0.6)	3.7 (0.6)	3.7 (0.6)	3.7 (0.6)	3.7 (0.6)	0.483
Energy, median (IQR), kcal/d	1,683.5 (1,250.8, 2,253.9)	1,081.0 (819.0, 1,373.9)	1,521.5 (1,250.8, 1,861.5)	1,872.0 (1,531.0, 2,278.0)	2,499.5 (2,021.8, 3,125.0)	<0.001
Protein, median (IQR), g/d	64.4 (46.0, 89.3)	40.0 (28.4, 52.4)	57.7 (46.1, 72.3)	72.7 (57.3, 91.4)	97.8 (77.6, 123.6)	<0.001
Carbohydrate, median (IQR), g/d	205.8 (148.8, 277.7)	129.7 (95.0, 173.6)	183.4 (148.3, 227.8)	226.9 (185.2, 278.4)	300.3 (239.2, 375.0)	<0.001
Total fat, median (IQR), g/d	61.9 (41.6, 89.4)	40.4 (26.9, 55.5)	57.3 (40.3, 77.4)	68.4 (49.6, 93.3)	93.5 (67.6, 126.9)	<0.001
Hypertension, n (%)	4,314 (53.2)	1,152 (57.1)	1,118 (55.5)	1,033 (50.6)	1,011 (49.9)	<0.001
Diabetes, n (%)	2,344 (28.9)	616 (30.5)	618 (30.7)	559 (27.4)	551 (27.2)	0.013
CHD, n (%)	900 (11.1)	239 (11.8)	227 (11.3)	226 (11.1)	208 (10.3)	0.459
Stroke, n (%)	767 (9.5)	257 (12.7)	202 (10.0)	167 (8.2)	141 (7.0)	<0.001
COPD history, n (%)	562 (6.9)	149 (7.4)	153 (7.6)	131 (6.4)	129 (6.4)	0.280
Cancer history, n (%)	1,364 (16.8)	324 (16.0)	349 (17.3)	340 (16.6)	351 (17.3)	0.654
G1	93 (1.1)	45 (2.2)	28 (1.4)	12 (0.6)	8 (0.4)	
G2	283 (3.5)	90 (4.5)	78 (3.9)	72 (3.5)	43 (2.1)	
G3	3,416 (42.2)	939 (46.5)	897 (44.5)	858 (42.0)	722 (35.6)	
G4	1,967 (24.3)	444 (22.0)	480 (23.8)	528 (25.8)	515 (25.4)	
G5	2,345 (28.9)	501 (24.8)	533 (26.4)	573 (28.0)	738 (36.4)	
ACR category, n (%)						<0.001
A1	2,530 (31.2)	656 (32.5)	669 (33.2)	664 (32.5)	541 (26.7)	
A2	4,594 (56.7)	1,097 (54.3)	1,099 (54.5)	1,167 (57.1)	1,231 (60.8)	
A3	980 (12.1)	266 (13.2)	248 (12.3)	212 (10.4)	254 (12.5)	
Mg intake, median (IQR), mg/d	235.0 (170.0, 323.0)	130.7 (105.0, 151.0)	202.0 (187.0, 218.0)	274.0 (254.0, 297.0)	399.0 (356.0, 473.0)	<0.001
Follow-up time, median (IQR), month	85.0 (44.0, 137.0)	86.0 (45.0, 139.0)	82.5 (44.0, 135.0)	87.0 (44.0, 136.0)	85.0 (45.0, 139.0)	0.566
All-cause mortality, n (%)	3,134 (38.7)	888 (44.0)	831 (41.2)	776 (38.0)	639 (31.5)	<0.001

Abbreviations: BMI, body mass index; SBP, systolic blood pressure; DBP, diastolic blood pressure; HGB, haemoglobin; HbA1c, haemoglobin A1c; Total Ca, total calcium; P, phosphorus; CHD, coronary heart disease; COPD, chronic obstructive pulmonary disease; eGFR, estimated glomerular filtration rate; ACR, albumin-to-creatinine ratio; Mg, magnesium.

a Through standard biochemistry testing.

The mean age was 63.9 years, 47.9% were male, the median dietary magnesium intake was 235.0 mg/day, and the median follow-up duration was 85 months. During follow-up, 3,134 participants (38.7%) died from all causes.

Participants were categorised into four dietary magnesium intake groups: Q1 (≤169.5 mg/day; n = 2,019), Q2 (170.0–234.0 mg/day; n = 2,016), Q3 (235.0–323.0 mg/day; n = 2,043), and Q4 (≥323.1 mg/day; n = 2,026). All-cause mortality decreased progressively from 44.0% in Q1–41.2% in Q2, 38.0% in Q3, and 31.5% in Q4 (P < 0.001). Participants with higher magnesium intake were generally younger, more likely to be male, had higher levels of physical activity, and were less likely to have hypertension or a history of stroke. The prevalence of COPD and cancer history did not differ significantly across quartiles.

### Associations between dietary magnesium intake and all-cause mortality

To investigate the association between dietary magnesium intake and all-cause mortality in CKD patients, multivariable Cox proportional hazards regression models were constructed with sequential adjustment for potential confounders (Table 2).

**Table 2:** Multivariable Cox regression models evaluating the association between dietary magnesium intake and all-cause mortality.

Dietary magnesium intake (mg/day)	Crude Model^a^		Model 1^b^		Model 2^c^		Model 3^d^		Model 4^e^	
	HR (95%CI)	*P-*value	HR (95% CI)	*P-*value	HR (95%CI)	*P-*value	HR (95%CI)	*P-*value	HR (95%CI)	*P-*value
Per 100 mg increase	0.896 (0.870–0.923)	<0.001	0.941 (0.912–0.971)	<0.001	0.953 (0.923–0.983)	0.002	0.922 (0.878–0.967)	<0.001	0.925 (0.881–0.971)	0.002
Quartile of magnesium intake										
Quartile 1 (≤169.5)	1(Reference)		1(Reference)		1(Reference)		1(Reference)		1(Reference)	
Quartile 2 (170.0–234.0)	0.970 (0.883–1.067)	0.533	0.973 (0.884–1.070)	0.571	0.985 (0.895–1.084)	0.753	0.973 (0.879–1.078)	0.602	0.978 (0.883–1.083)	0.673
Quartile 3 (235.0–323.0)	0.886 (0.805–0.976)	0.014	0.898 (0.813–0.991)	0.033	0.923 (0.836–1.020)	0.115	0.902 (0.804–1.013)	0.082	0.904 (0.804–1.016)	0.090
Quartile 4 (≥323.1)	0.719 (0.650–0.796)	<0.001	0.849 (0.763–0.944)	0.002	0.893 (0.803–0.994)	0.039	0.876 (0.753–1.018)	0.085	0.866 (0.744–1.007)	0.062
P for trend										0.002

Abbreviations: HbA1c, glycated haemoglobin; eGFR, estimated glomerular filtration rate; UACR, albumin-to-creatinine ratio; HR, hazard ratio; CI, confidence interval.

a Crude model: unadjusted.

b Model 1: adjusted for age, sex, marital status, race/ethnicity, educational level, and family income.

c Model 2: additionally adjusted for body mass index, smoking status, drinking status, physical activity, systolic blood pressure, and diastolic blood pressure.

d Model 3: additionally adjusted for haemoglobin, glycated haemoglobin, serum calcium, serum phosphorus, protein intake, total energy intake, carbohydrate intake, and total fat intake.

e Model 4: additionally adjusted for hypertension, diabetes, coronary heart disease, stroke, chronic obstructive pulmonary disease, cancer history, eGFR category, and UACR category.

When dietary magnesium intake was analysed as a continuous variable, higher magnesium intake was consistently associated with a lower risk of all-cause mortality across all models. In the crude model, each 100 mg/day increase in dietary magnesium intake was associated with a 10.4% lower risk of all-cause mortality (HR 0.896, 95% CI 0.870–0.923; P < 0.001). This association remained significant after sequential adjustment for demographic characteristics, lifestyle factors, dietary variables, clinical indicators, kidney disease severity, COPD, and cancer history. In the fully adjusted model (Model 4), each 100 mg/day increase in dietary magnesium intake was associated with a 7.5% lower risk of all-cause mortality (HR 0.925, 95% CI 0.881–0.971; P = 0.002).

When dietary magnesium intake was categorised into quartiles, the point estimates suggested a progressive reduction in mortality risk with increasing magnesium intake. Compared with participants in Quartile 1 (≤169.5 mg/day), the fully adjusted hazard ratios were 0.978 (95% CI 0.883–1.083; P = 0.673) for Quartile 2, 0.904 (95% CI 0.804–1.016; P = 0.090) for Quartile 3, and 0.866 (95% CI 0.744–1.007; P = 0.062) for Quartile 4. Although none of the individual quartile comparisons reached conventional statistical significance after full adjustment, a significant inverse trend across quartiles was observed (P for trend = 0.036), indicating that higher dietary magnesium intake was generally associated with a lower risk of all-cause mortality.

To further evaluate the dose–response relationship between dietary magnesium intake and all-cause mortality, restricted cubic spline (RCS) analysis was performed. A significant overall inverse association was observed between dietary magnesium intake and all-cause mortality (P for overall association = 0.009), with no evidence of nonlinearity (P for nonlinearity = 0.472) (Fig. 4).

**Fig. 2:**
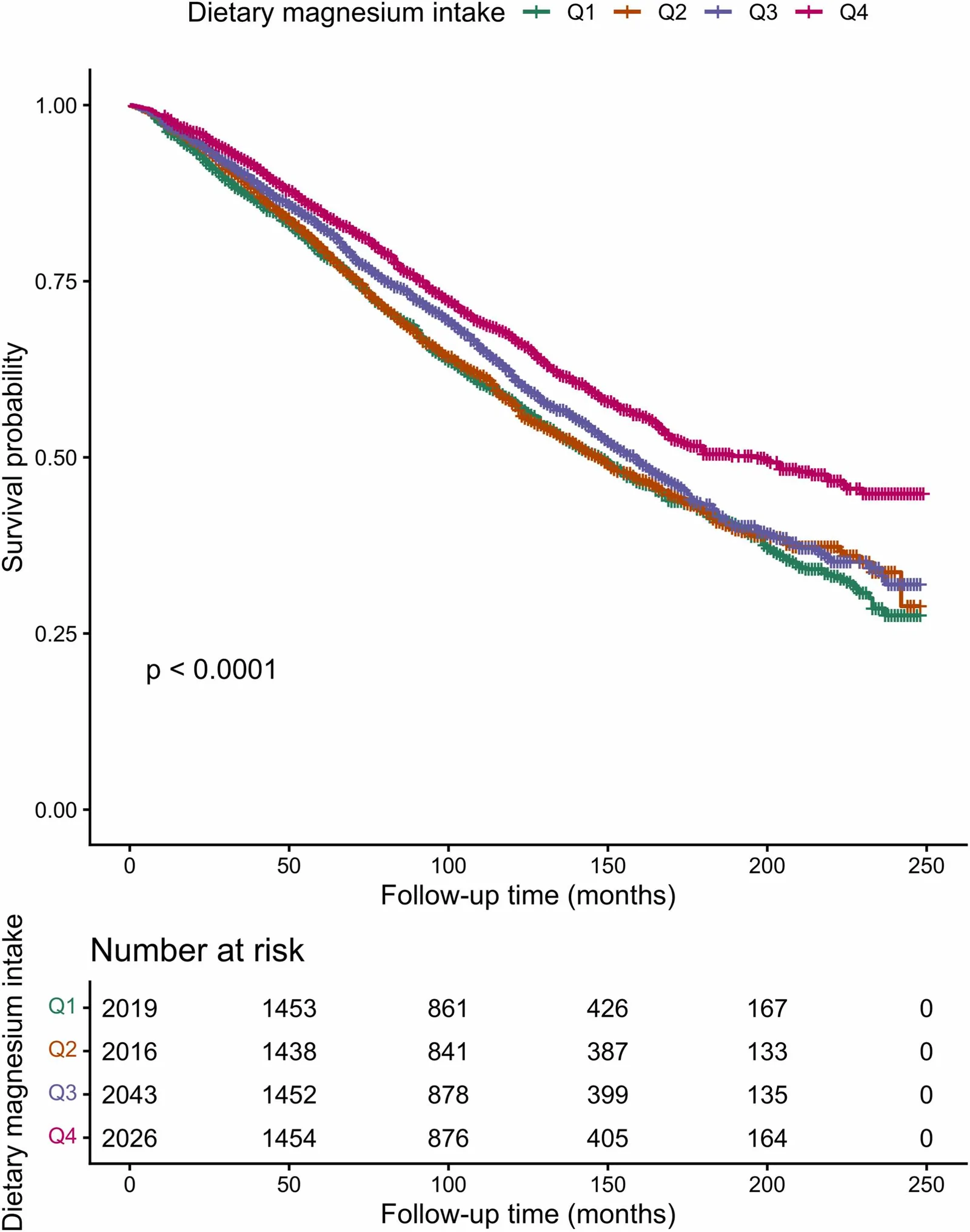
Kaplan–Meier survival curves for all-cause mortality according to quartiles of dietary magnesium intake among adults with chronic kidney disease. Differences were assessed using the log-rank test. The numbers at risk are shown below the curves.

Additionally, Kaplan–Meier survival analysis demonstrated significant differences in survival probabilities across the four quartiles of dietary magnesium intake (log-rank P < 0.0001), with participants in the highest quartile (Q4) exhibiting the highest survival probability during follow-up (Fig. 2).

### Subgroup analyses

**Fig. 3:**
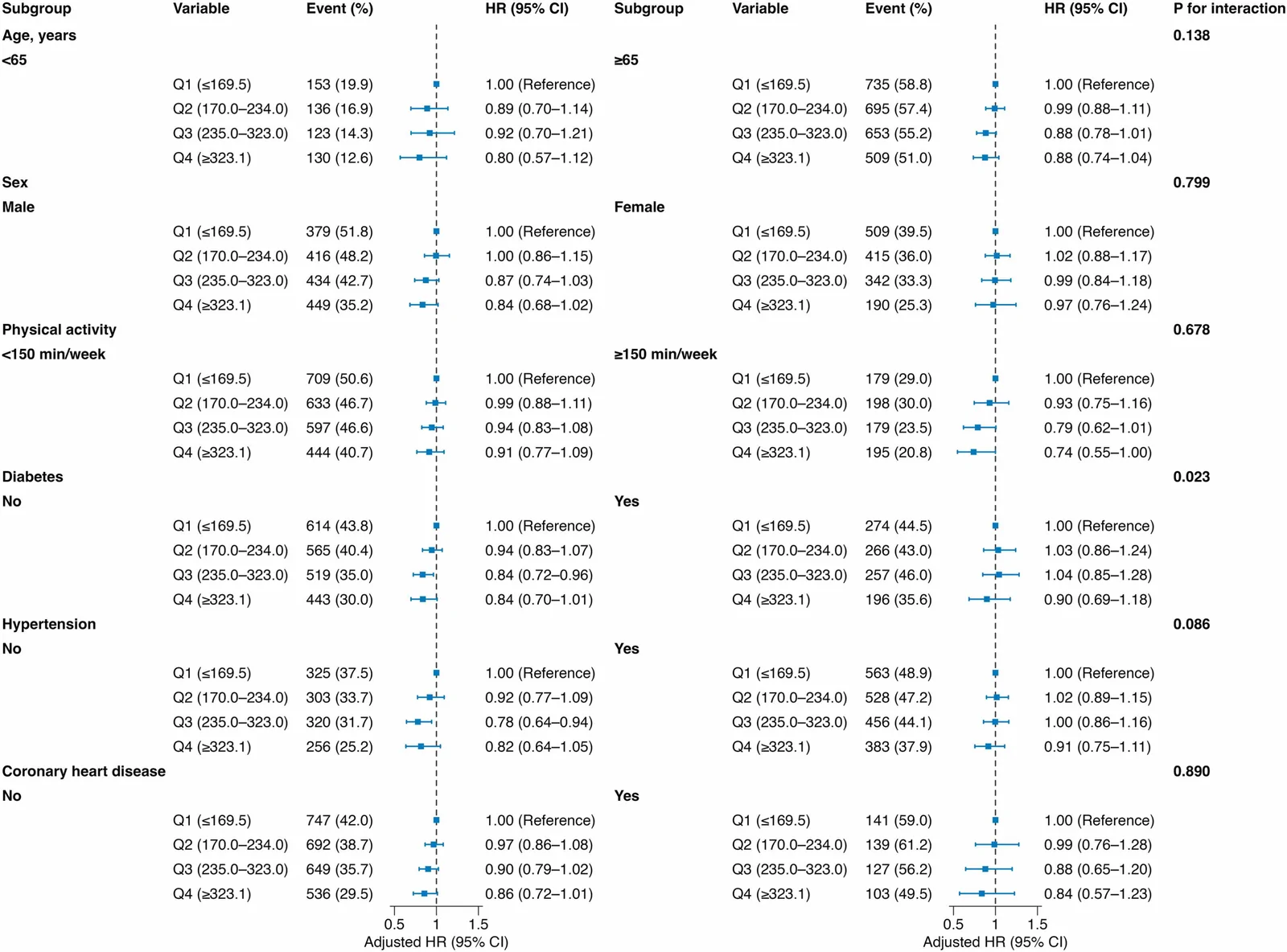
Subgroup analysis of the association between dietary magnesium intake and all-cause mortality. The model was adjusted for age, sex, race/ethnicity, educational level, marital status, family income, smoking status, drinking status, physical activity, body mass index, systolic blood pressure, diastolic blood pressure, haemoglobin, HbA1c, serum total calcium, serum phosphorus, energy intake, carbohydrate intake, protein intake, total fat intake, hypertension, diabetes, coronary heart disease, stroke, COPD, cancer history, eGFR category, and UACR category, excluding the stratification factor itself. Abbreviations: CHD, coronary heart disease; HR, hazard ratio; CI, confidence interval.

We performed subgroup analyses to assess effect modification in the relationship between dietary magnesium intake and all-cause mortality, stratified by age, sex, physical activity, diabetes, hypertension, and coronary heart disease (Fig. 3). Overall, the inverse association between dietary magnesium intake and all-cause mortality was generally consistent across the predefined subgroups. A statistically significant interaction was observed for diabetes status (P for interaction = 0.023), whereas no significant interactions were detected for age (P = 0.138), sex (P = 0.799), physical activity (P = 0.678), hypertension (P = 0.086), or coronary heart disease (P = 0.890). Considering the exploratory nature of these subgroup analyses and the multiple comparisons performed, the observed interaction for diabetes should be interpreted with caution and requires confirmation in future studies.

**Fig. 4:**
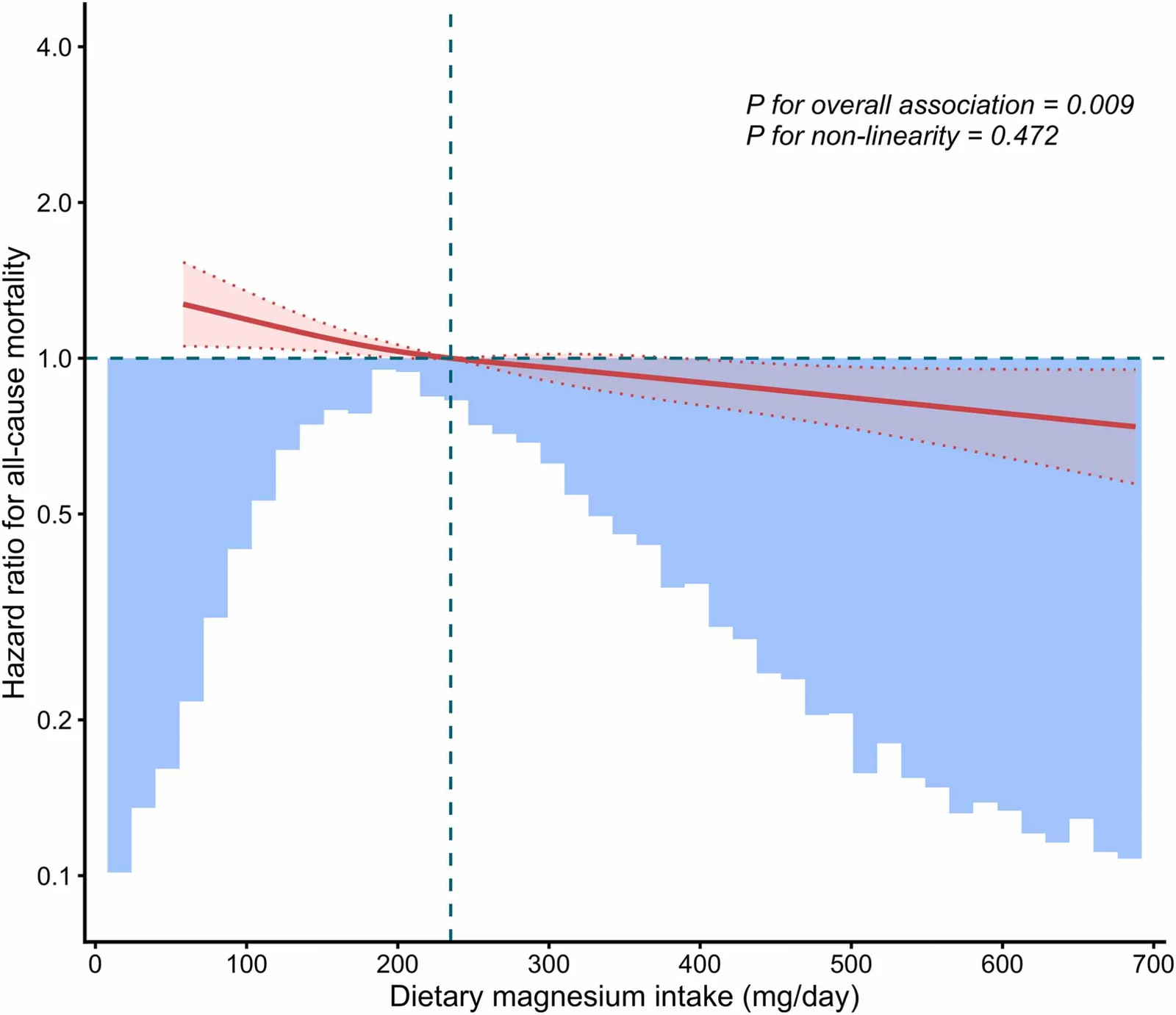
Restricted cubic spline analysis of the association between dietary magnesium intake and all-cause mortality among adults with chronic kidney disease. The solid red line represents the adjusted hazard ratio (HR), and the red dotted lines represent the corresponding 95% confidence interval (CI). The median dietary magnesium intake of 235 mg/day was used as the reference value (HR = 1.00), as indicated by the vertical dashed line; the horizontal dashed line indicates an HR of 1.00. The histogram at the bottom shows the distribution of dietary magnesium intake. The restricted cubic spline model was fitted with four knots and adjusted for age, sex, race/ethnicity, educational level, marital status, family income, smoking status, drinking status, physical activity, body mass index, systolic blood pressure, diastolic blood pressure, haemoglobin, HbA1c, serum total calcium, serum phosphorus, energy intake, carbohydrate intake, protein intake, total fat intake, hypertension, diabetes, coronary heart disease, stroke, chronic obstructive pulmonary disease, cancer history, eGFR category, and UACR category. The overall association was statistically significant (P for overall association = 0.009), with no evidence of a nonlinear association (P for nonlinearity = 0.472).

### Sensitivity analyses

Sensitivity analyses yielded findings consistent with the primary analysis (Table 3). The complete-case analysis included all 8,104 participants and produced an adjusted HR of 0.925 (95% CI 0.881–0.971; p = 0.002) per 100 mg/day increase in dietary magnesium intake. After excluding deaths within the first two years of follow-up, the association was attenuated but remained significant (HR 0.943, 95% CI 0.895–0.993; p = 0.026). Similar results were observed after excluding participants with extreme energy intake (HR 0.925, 95% CI 0.880–0.972; P = 0.002) and participants identified as receiving dialysis (HR 0.925, 95% CI 0.881–0.971; p = 0.002).

**Table 3:** Sensitivity analyses of the association between dietary magnesium intake and all-cause mortality.

Dietary magnesium intake (mg/day)	Deaths, n/N	Crude Model		Adjusted Model	
		HR (95%CI)	*P-*value	HR (95%CI)	*P-*value
Excluding participants with missing covariate data					
Per 100 mg/day increase	3,134/8,104	0.896 (0.870–0.923)	<0.001	0.925 (0.881–0.971)	0.002
Excluding participants who died within the first 2 years of follow-up					
Per 100 mg/day increase	2,632/7,602	0.910 (0.882–0.939)	<0.001	0.943 (0.895–0.993)	0.026
Excluding participants with extreme energy intake^a^					
Per 100 mg/day increase	3,079/7,960	0.894 (0.867–0.922)	<0.001	0.925 (0.880–0.972)	0.002
Excluding participants identified as receiving dialysis					
Per 100 mg/day increase	3,093/8,025	0.899 (0.873–0.925)	<0.001	0.925 (0.881–0.971)	0.002

Crude Model: Unadjusted.

Adjusted Model: Adjust for age, sex, race/ethnicity, education level, marital status, family income, smoking status, drinking status, physical activity, body mass index, systolic blood pressure, diastolic blood pressure, haemoglobin, HbA1c, serum total calcium, serum phosphorus, energy intake, carbohydrate intake, protein intake, total fat intake, hypertension, diabetes, coronary heart disease, stroke, eGFR category and UACR category.

Abbreviations: HbA1c, haemoglobin A1c; eGFR, estimated glomerular filtration rate; ACR, albumin-to-creatinine ratio; CI, confidence interval; HR, hazard ratio.

a Energy intake less than 500 kcal or more than 5,000 kcal per day.

## Discussion

To the best of our knowledge, this study is among the first large-scale cohort analyses to examine the association between dietary magnesium intake and all-cause mortality among adults with CKD. Higher dietary magnesium intake was associated with a lower risk of all-cause mortality when magnesium intake was analysed as a continuous exposure. This association remained significant after adjustment for demographic characteristics, lifestyle factors, dietary intake, clinical indicators, kidney disease severity, COPD, and cancer history, and was generally supported by the sensitivity analyses. Quartile-based estimates showed a similar inverse pattern, although the comparison between the highest and lowest quartiles did not reach conventional statistical significance after full adjustment. Nevertheless, the significant trend across quartiles and the restricted cubic spline findings support an overall inverse association between dietary magnesium intake and mortality risk.

In the United States, the recommended daily intake of magnesium is 400 mg for men aged 19–30, 420 mg for males aged 30 and above, 310 mg for women aged 19–30, and 320 mg for females aged 30 and above.^24^ The 2015–2020 dietary guidelines for Americans identify magnesium as one of the most commonly deficient nutrients.^25^ A study based on NHANES data from 2001 to 2008 found that more than half of U.S. adults do not meet the recommended intake for magnesium, with an average intake of only 275–300 mg/day.^26^ Similarly, in our study, the median dietary magnesium intake among CKD patients was 235 mg/day, which is well below the recommended intake for both men and women.

Previous studies have shown that hypomagnesaemia is significantly associated with an increased risk of mortality in both dialysis and pre-dialysis chronic kidney disease (CKD) patients.^27^, ^28^, ^29^, ^30^ Magnesium is primarily obtained through the diet, with magnesium-rich foods such as leafy vegetables, whole grains, and nuts. Recent studies have indicated that increasing dietary magnesium intake may improve mortality risk in patients with various conditions.^31^ In a meta-analysis of 19 prospective cohort studies by Bagheri *et al*., a 100 mg/day increase in dietary magnesium intake was associated with a 6% reduction in all-cause mortality risk.^32^ In our fully adjusted model, each 100 mg/day increment in dietary magnesium intake was associated with a 7.5% lower risk of all-cause mortality among adults with CKD (HR 0.925, 95% CI 0.881–0.971). A similar inverse association has also been reported among individuals with rheumatoid arthritis, in whom higher dietary magnesium intake was associated with reduced all-cause mortality.^13^ Taken together, these findings suggest that dietary magnesium may be associated with survival across different populations with chronic disease. However, findings from previous studies have not been entirely consistent. A cohort study of stroke survivors reported that total magnesium intake, including supplemental intake, was associated with lower all-cause mortality, whereas dietary magnesium intake alone was not significantly associated with mortality.^33^ Similarly, a prospective study of Swedish men found no significant association between dietary magnesium intake and all-cause, cardiovascular, or cancer mortality.^34^ Differences in baseline magnesium intake, dietary patterns, study populations, comorbidity burden, exposure assessment, and adjustment strategies may partly explain these inconsistencies. Participants in the Swedish cohort generally had substantially higher magnesium intake than those in the present study, suggesting that the potential benefit associated with increasing magnesium intake may be more apparent in populations with relatively low baseline intake. Importantly, the exposure assessed in the present study was food-derived dietary magnesium rather than total magnesium intake or supplemental magnesium. Therefore, our findings should not be directly extrapolated to magnesium supplementation and should not be interpreted as evidence supporting routine magnesium supplementation in patients with CKD.

When dietary magnesium intake was categorised into quartiles, mortality risk showed a generally decreasing pattern across increasing intake levels. Compared with Q1, the fully adjusted HRs were 0.978 for Q2, 0.904 for Q3, and 0.866 for Q4. Although the individual quartile comparisons did not reach conventional statistical significance, the trend across quartiles was significant. This discrepancy between the significant continuous association and the non-significant individual quartile comparisons may reflect the loss of statistical information caused by categorising a continuous exposure. Categorisation reduces within-group variability and may reduce statistical power, particularly after adjustment for dietary factors that are strongly correlated with magnesium intake, including total energy, protein, carbohydrate, and fat intake. The restricted cubic spline analysis provided further support for an inverse dose–response association. Dietary magnesium intake was significantly associated with all-cause mortality overall, whereas there was no statistically significant evidence of nonlinearity.

This suggests that the association was approximately linear across the observed intake range rather than characterised by a clear threshold, plateau, or U-shaped pattern. The wider confidence intervals at the lower and upper extremes of magnesium intake likely reflect the smaller numbers of participants in these exposure ranges. Therefore, the spline results should be interpreted primarily within the central range of the observed magnesium distribution.

Subgroup analyses suggested that the inverse association was generally observed across most predefined subgroups. A statistically significant interaction was identified for diabetes status, whereas no significant interactions were observed for age, sex, physical activity, hypertension, or coronary heart disease. Among participants without diabetes, higher dietary magnesium intake was associated with lower mortality risk, whereas the association was weaker among participants with diabetes. Several potential explanations may be considered. Diabetes is associated with altered magnesium metabolism, increased urinary magnesium loss, insulin resistance, medication use, and greater comorbidity burden, which may attenuate the association between dietary intake and mortality. Nevertheless, because the subgroup analyses were exploratory and involved multiple comparisons, the interaction with diabetes should be interpreted cautiously and requires confirmation in independent studies.

Several biological mechanisms may explain the inverse association between dietary magnesium intake and mortality among patients with CKD. Magnesium has anti-inflammatory, antioxidant, and vascular protective properties. Chronic inflammation and oxidative stress contribute to CKD progression, cardiovascular complications, and increased mortality.^35^ Magnesium intake has been found to be inversely correlated with various inflammatory markers, including C-reactive protein and interleukin-6, suggesting its potential anti-inflammatory effect.^36^, ^37^ In CKD animal models, dietary magnesium supplementation was shown to significantly decrease levels of inflammatory cytokines such as TNF-α, IL-1β, and IL-6, as well as oxidative stress markers like glutathione peroxidase (GPx), thereby mitigating CKD-related inflammation and oxidative damage.^38^ Furthermore, magnesium inhibited the activation of the NF-κB pathway, which led to reduced production of reactive oxygen species (ROS) and effectively reduced cytokine upregulation in inflammatory conditions.^14^ Magnesium’s role in regulating inflammatory signalling has been demonstrated in both healthy animals and CKD models.^39^

Additionally, high-magnesium diets have been found to reduce lipid peroxidation, reinforcing its antioxidant effects.^40^ In uraemic rats, increased magnesium intake significantly reduced vascular calcification and mortality, suggesting that magnesium may improve CKD prognosis by regulating calcium metabolism and oxidative stress.^14^ These mechanisms provide biological plausibility for the observed association, although the present observational study cannot determine which pathways were primarily responsible.

This study has several strengths. It included a relatively large sample of adults with CKD, long-term mortality follow-up, detailed information on demographic, lifestyle, dietary, laboratory, and clinical characteristics, and multiple complementary analytical approaches. Dietary magnesium was examined as both a continuous and categorical exposure, and the dose–response relationship was evaluated using restricted cubic splines. In addition, the results were assessed through subgroup and sensitivity analyses.

Several limitations should also be acknowledged. First, because of the observational design, causal inference cannot be established, and residual or unmeasured confounding remains possible. Second, NHANES provides kidney function and albuminuria measurements from a single examination. Therefore, CKD status could not be confirmed using repeated measurements over at least three months, as required in clinical practice. Third, dietary magnesium intake was based on a single 24-h dietary recall, which may not fully reflect long-term habitual intake and is subject to recall and reporting errors. Fourth, dietary intake was assessed only at baseline, and changes in magnesium intake during follow-up could not be evaluated.

Fifth, serum magnesium concentrations were unavailable in the current analytic dataset. Therefore, we were unable to evaluate the correlation between dietary magnesium intake and circulating magnesium status or determine whether serum magnesium mediated the observed association. Sixth, information on some clinical factors, including kidney transplantation status and detailed medication use, was unavailable. Seventh, the proportional hazards assumption was not fully satisfied for the global Cox model, although no evidence of non-proportionality was observed for the dietary magnesium exposure variable itself. The reported hazard ratios should therefore be interpreted as average associations over the follow-up period. Finally, the study population consisted of U.S. adults with CKD, and the generalisability of these findings to populations with different dietary patterns, healthcare systems, or ethnic backgrounds requires further investigation.

## Conclusion

In this large prospective cohort of adults with chronic kidney disease, higher dietary magnesium intake was independently associated with a lower risk of all-cause mortality. This inverse association remained robust across multiple sensitivity analyses and was supported by dose–response analyses, suggesting that adequate dietary magnesium intake may be an important nutritional factor associated with prognosis in patients with CKD. Further prospective studies and randomised clinical trials are warranted to determine whether increasing dietary magnesium intake can improve clinical outcomes in this population.

## CRediT authorship contribution statement

**Li-jun Xie:** Data curation. **Li Zhang:** Funding acquisition. **Jing Zou:** Writing – original draft. **Mei-zhu Gao:** Formal analysis. **Yan-feng Zhou:** Validation. **Zi-yan Xu:** Writing – original draft. **Dan-dan Ruan:** Resources. **Bin Hu:** Funding acquisition. **Ruo-li Wang:** Visualization. **Xiao-lan Wang:** Validation. **Fang-meng Huang:** Supervision. **Juan Zhu:** Formal analysis. **Ying Ye:** Conceptualization. **Yun-fei Li:** Supervision. **Qian Chen:** Methodology. **Li Chen:** Funding acquisition. **Zhu-ting Fang:** Supervision. **Zhi-hai Zheng:** Funding acquisition. **Jian-hui Zhang:** Validation. **Kai-ying He:** Data curation, Writing – original draft. **Jie-wei Luo:** Writing – review & editing, Supervision. **Yi-jia Luo:** Investigation.

## Ethics approval and consent to participate

Study protocols for NHANES were approved by the NCHS ethnics review board (Protocol #2011-17, https://www.cdc.gov/nchs/nhanes/irba98.htm). All the participants signed the informed consent before participating in the study. All methods were carried out in accordance with relevant guidelines and regulations.

## Funding

This work was supported by the Fujian Province Natural Science Fund Project (2026J001171, 2026J001172, 2024J011017, 2024Y0033, 2024J08250, 2025J08038), Joint Funds for the Innovation of Science and Technology in Fujian Province (2025Y9011, 2025Y9008, 2024Y9081, 2025Y9087), the Fujian Provincial Health Commission Science and Technology Program (2024QNA002, 2024GGA090), the Special Research Foundation of Fujian Provincial Department of Finance (2025-0929#，2025-876#), Fujian Provincial Senior Talent Training Progam on Western Medicine Doctors Learning from Traditional Chinese Medicine (Zhi-hai Zheng, Meng Lin), and National famous and old Chinese medicine experts (Hong Li, Chunjin Yi, Shaoguang Lv) inheritance studio construction project, China.

## Declaration of competing interest

All authors declare that they have no competing interests.

## Data Availability

These survey data are free and publicly available, and can be downloaded directly from the NHANES website (http://www.cdc.gov/nchs/nhanes.htm) by users and researchers worldwide.
